# Supplementary figures and images for: ULK1 drives NDP52-mediated selective autophagic degradation of MHC-I to promote immune evasion in HPV-positive head and neck cancer
Source: bioRxiv. 2026 Mar 17:2026.03.14.711071. Preprint. [Version 1] doi: 10.64898/2026.03.14.711071 (PMC13015544; doi:10.64898/2026.03.14.711071)

Fig S1

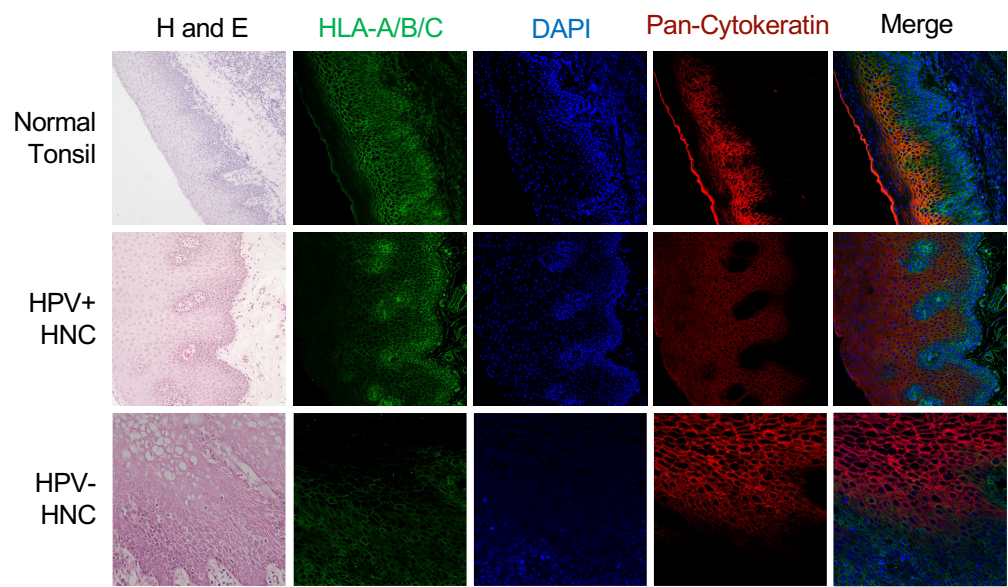

Fig S2

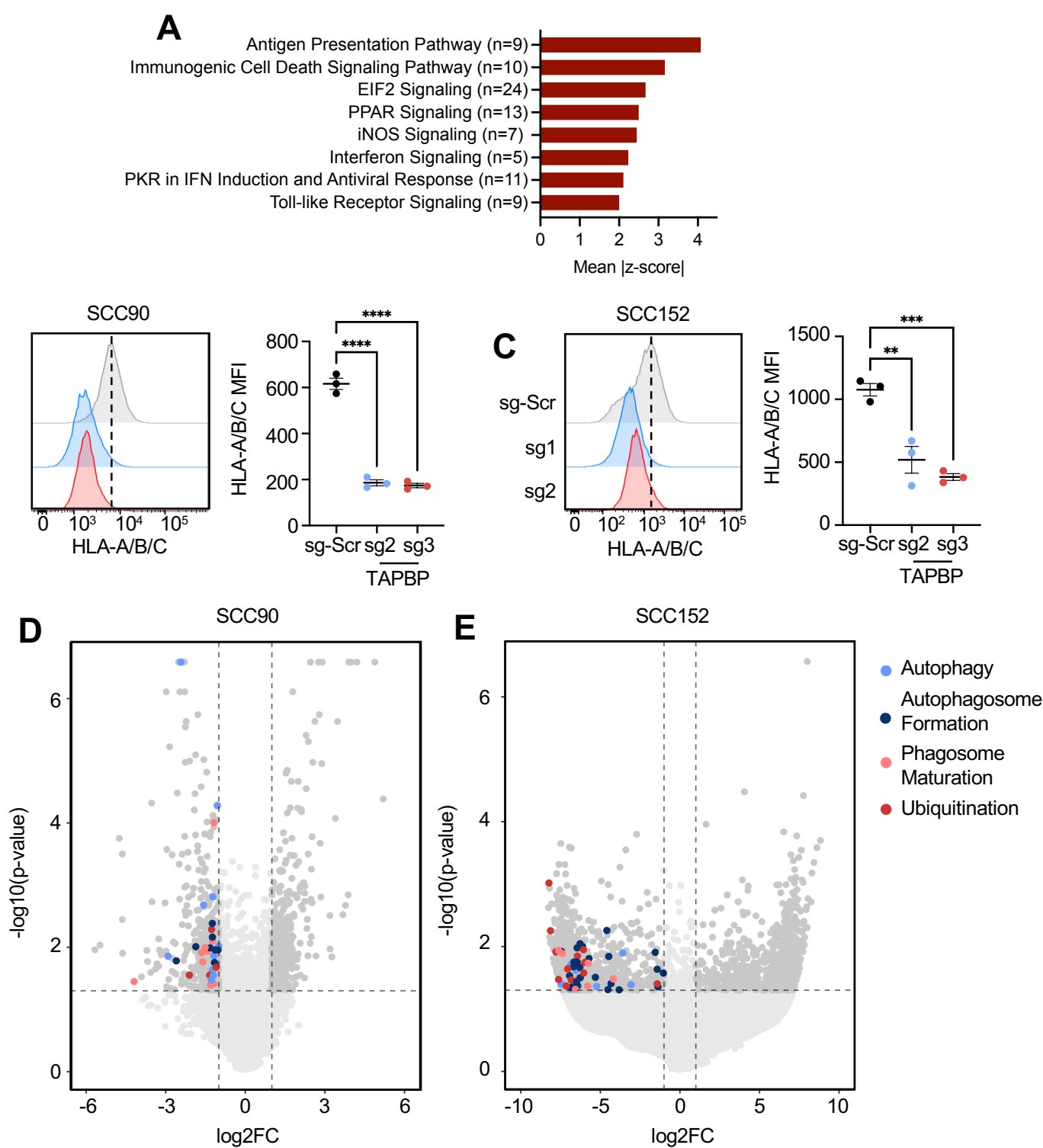

**Fig S3**

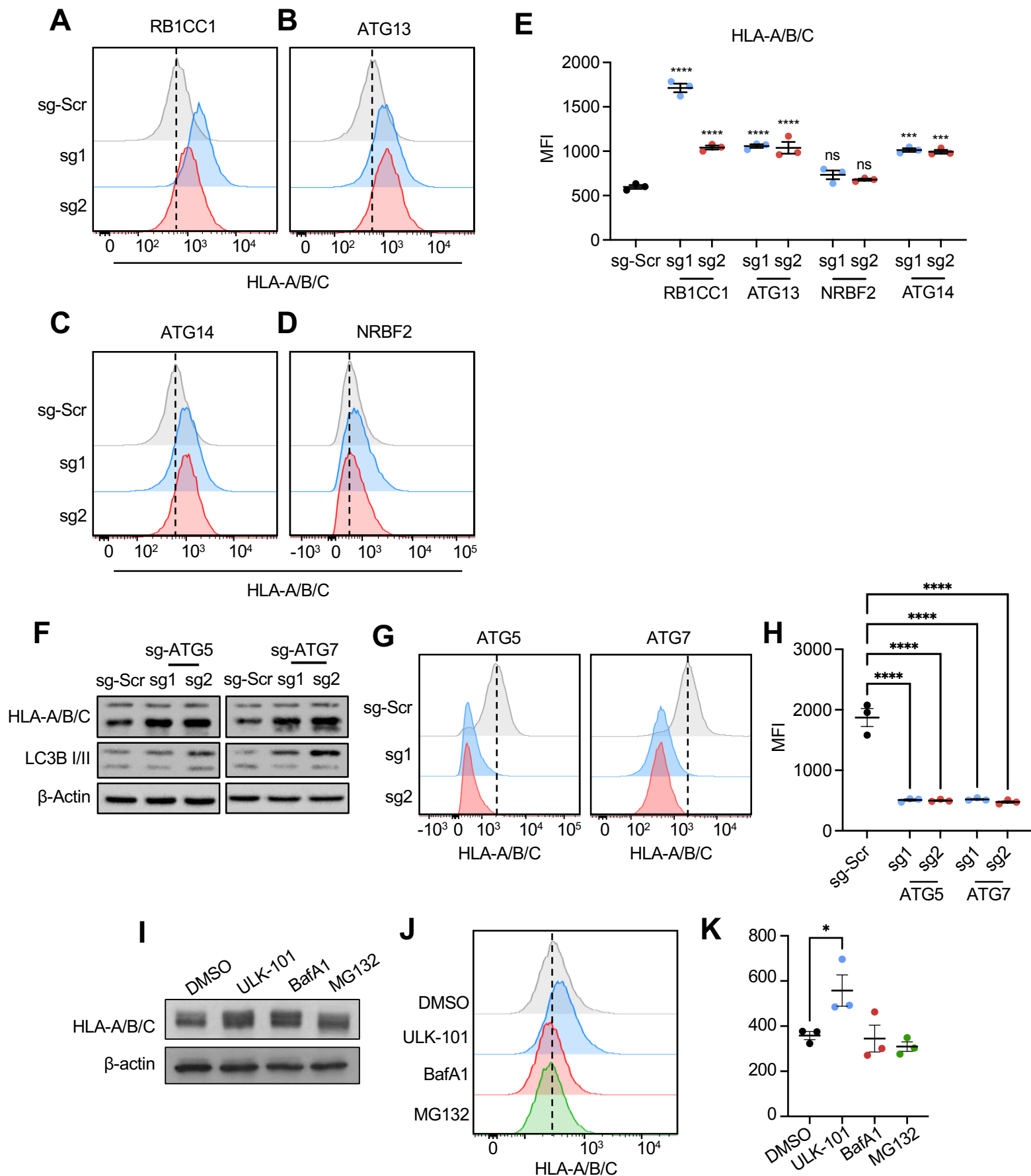

**Fig S4**

**A**

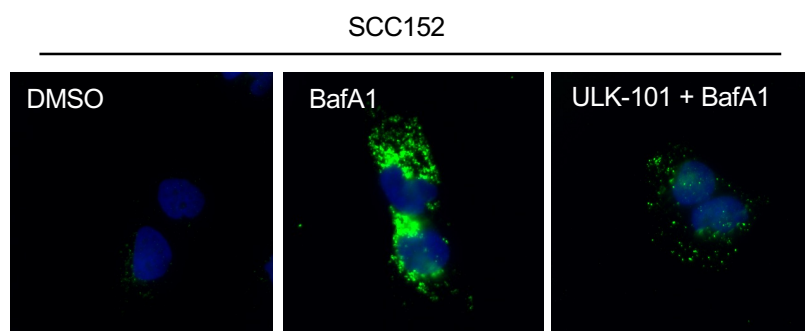

**B**

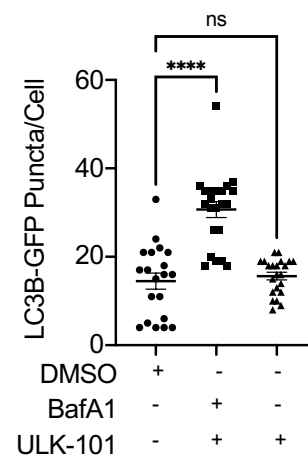

**C**

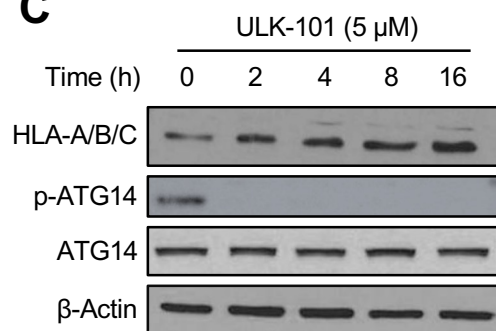

**D**

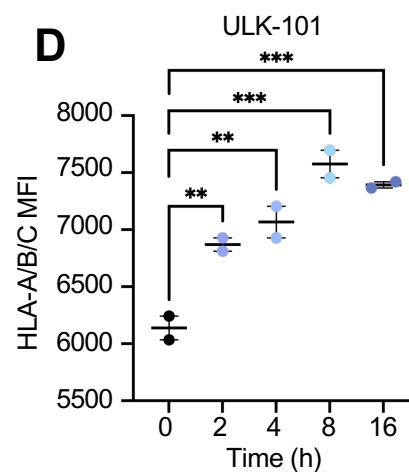

**E**

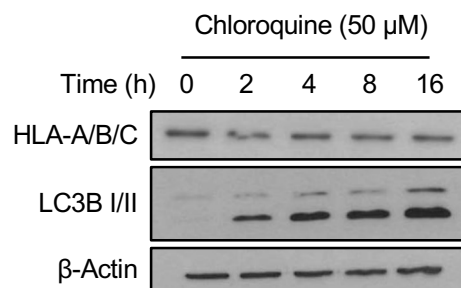

**F**

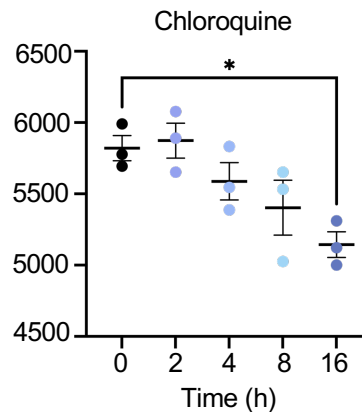

**G**

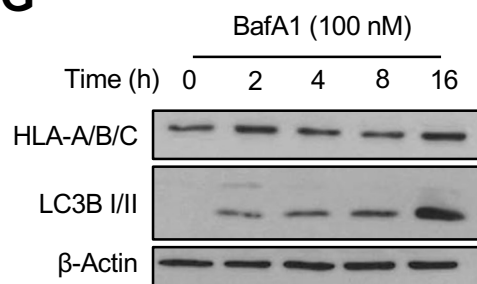

**H**

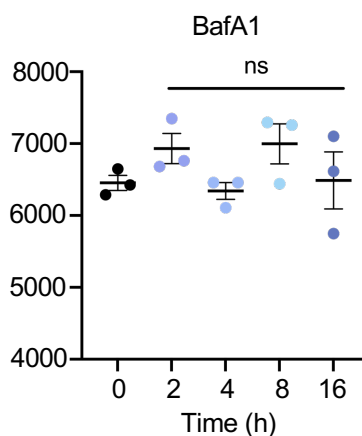

**Fig S5**

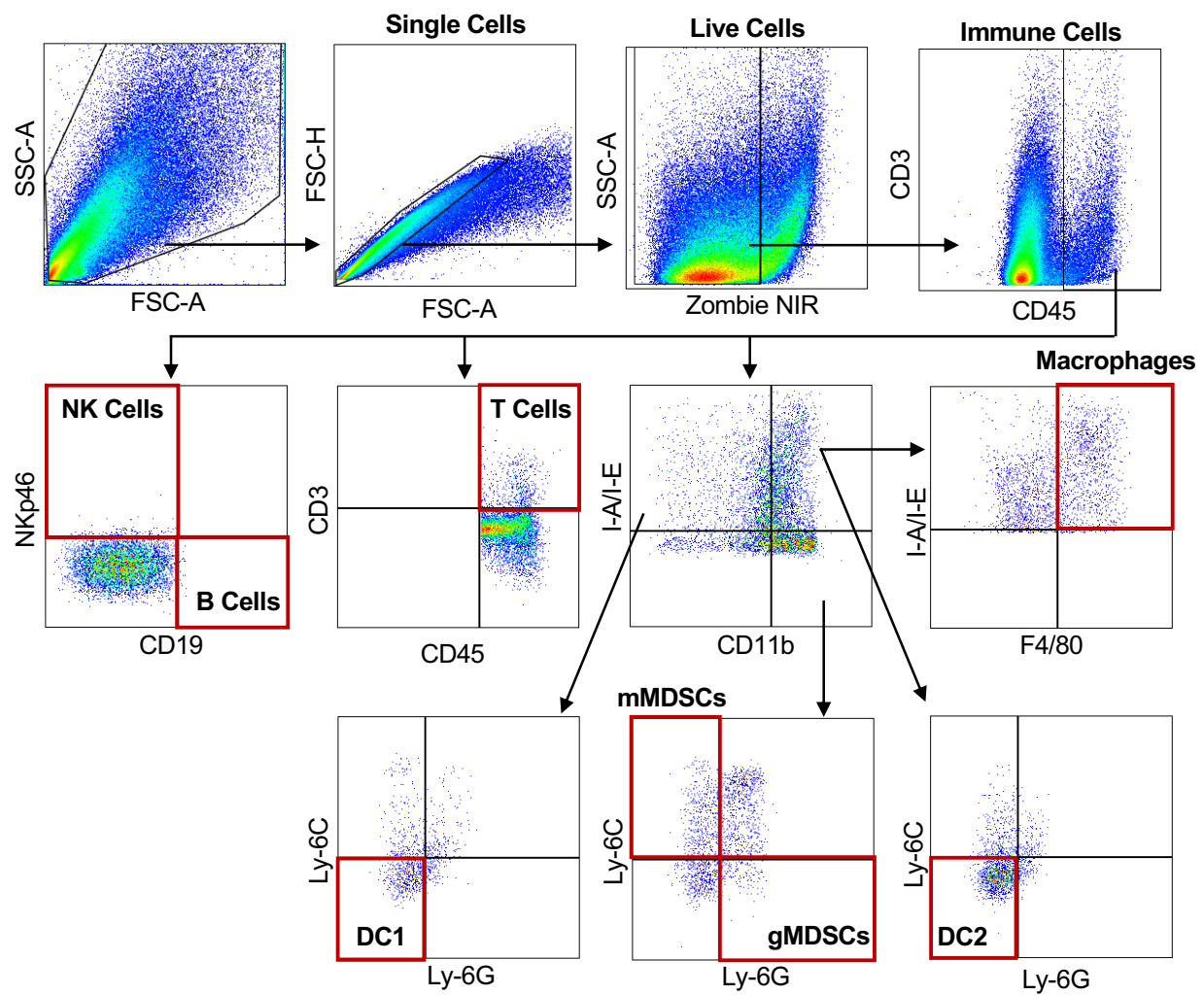

**Fig S6**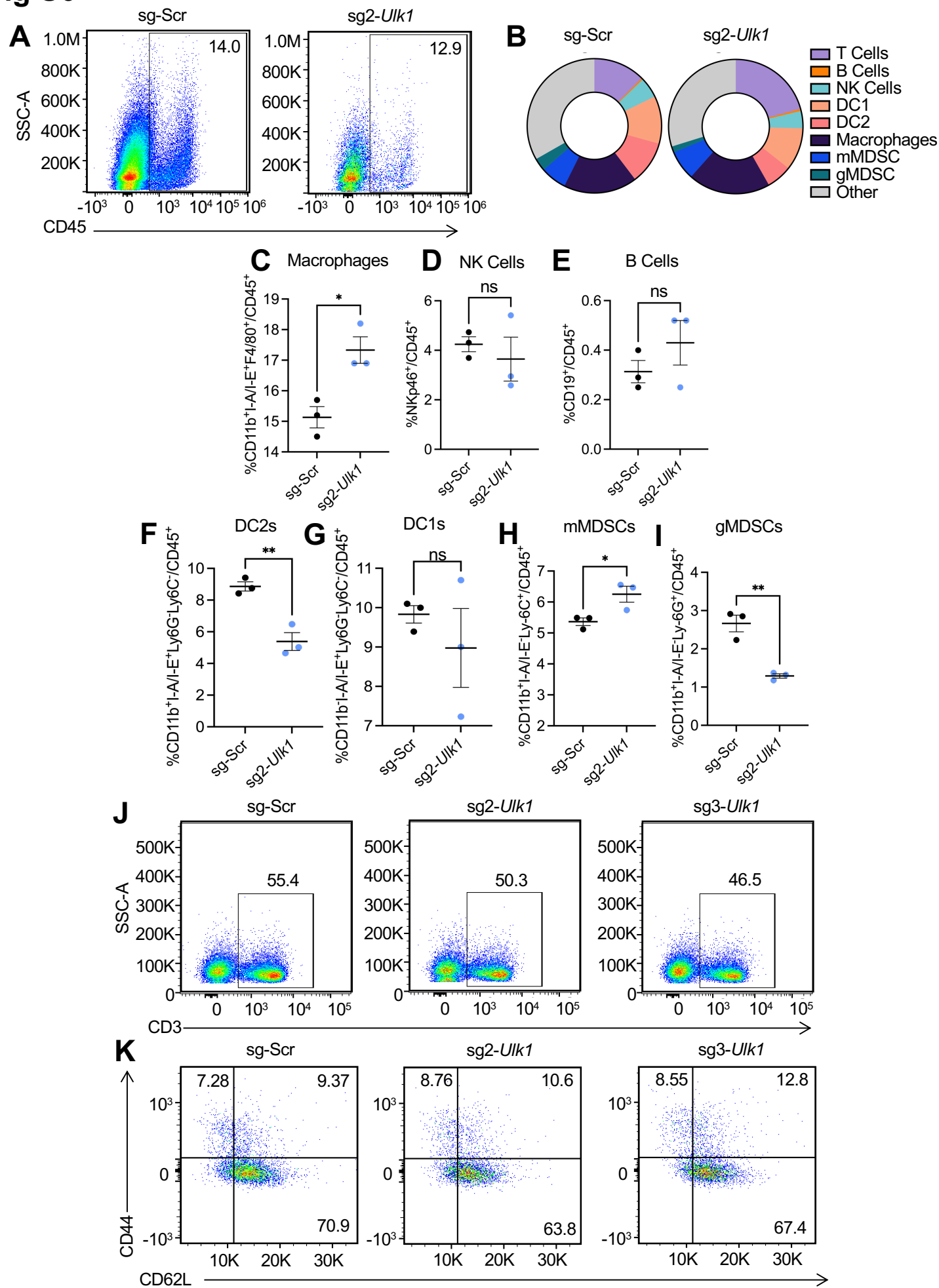

Supplement: Supplement 1 — Fig. S1. MHC-I protein levels are decreased in HPV+ and HPV-negative patient tumors. Tissue sections of normal tonsil (n=16), HPV+ HNCs (n=23), and HPV− HNCs (n=11) were stained with hematoxylin & eosin, anti-HLA-A/B/C (green), anti-pan-cytokeratin (red), and DAPI (blue). Representative images are shown. Fig. S2. Genome-wide CRISPR screens identify key regulators of cell-surface MHC-I expression on HPV+ HNC cells. Pathways enriched in the significant positive regulators (p-value <0.05 and |Log2FC| >1) from the genome-wide CRISPR screens were identified using Ingenuity Pathway Analysis (IPA) (A). Screens were validated by knocking out tapasin (TAPBP) and evaluating HLA-A/B/C expression by flow cytometry (B and C). Volcano plots showing gene hits from the autophagy, autophagosome formation, phagosome maturation, and ubiquitination pathways in SCC90 (D) and SCC152 (E) cells, as identified from Ingenuity Pathway Analysis. Fig. S3. Inhibition of autophagy initiation, but not post-initiation, restores surface MHC-I expression in HPV+ HNC cells. Genes in the ULK1 (ATG13 and RB1CC1) and PIK3C3 (ATG14 and NRBF2) autophagy initiation complexes that were significant negative regulators in the CRISPR screen were knocked out of SCC90 cells. MHC-I surface expression was determined by flow cytometry (A – E). Genes involved in autophagosome formation (ATG5 and ATG7) were knocked out in SCC90 cells. Inhibition of autophagosome formation was assessed by western blotting with antibodies against LC3B-I/II (F). Total (F) and cell-surface (G) MHC-I levels were determined by western blotting and flow cytometry, respectively. SCC90 cells were then treated with inhibitors against autophagy initiation (5 μM ULK-101), post-initiation (100 nM Bafilomycin A1), and the proteasome (10 μM MG132) for 16 h. Total and cell-surface MHC-I levels were determined by western blotting (H) and flow cytometry (I and J), respectively. P-value was determined by One-way ANOVA. *p < 0.05, **p < 0.01, ***p < 0.00 [file media-1.pdf]
