# Supplementary material for: ULK1 drives NDP52-mediated selective autophagic degradation of MHC-I to promote immune evasion in HPV-positive head and neck cancer
Source: bioRxiv. 2026 Mar 17:2026.03.14.711071. Preprint. [Version 1] doi: 10.64898/2026.03.14.711071 (PMC13015544; doi:10.64898/2026.03.14.711071)
Supplement: Supplement 2 [file media-2.pdf]

Table S1. Patient tumor sample information.

Sample ID	HPV Status	Site	AJCC Edition	TNM Stage	Stage	Procedure	Treatment
DCA 019-1048	HPV positive	Tonsil	7	cT2N2b, pT2NX	IVa	Resection	Untreated
DCA 022-1035	HPV positive	Tonsil	7	cT2N1	III	Biopsy	Untreated
DCA 029-1250	HPV positive	Tonsil	7	cT3N2b, pT2NX	IVa	Resection	Untreated
DCA 031-1296	HPV positive	Tonsil	7	cT2N2b, pT2NX	IVa	Resection	Untreated
DCA 036-1223	HPV positive	Tonsil	7	cT2N2c	IVa	Biopsy	Untreated
DCA 038-1165	HPV positive	BOT	7	cT1N2a	IVa	Biopsy	Untreated
DCA 043-1253	HPV positive	Tonsil	7	cT4aN2b	IVa	Biopsy	Untreated
DCA FG 1124	HPV positive	Tonsil	7	cT1N2a, pT1NX	IVa	Biopsy	Untreated
DCA FG 1140	HPV positive	Tonsil	7	cT1N2a, pT1NX	IVa	Biopsy	Untreated
HN 006	HPV positive	BOT	6	cT3N2b	IVa	Biopsy	Untreated
HN 011	HPV positive	Tonsil	6	cT2N2b	IVa	Biopsy	Untreated
HN 017	HPV positive	R. tonsil	6	cT2N2b, pT2N2b	IVa	Salvage neck dissection	Post-CRT
HN 033	HPV positive	Oropharynx	6	cT4N2c	IVa	Biopsy	Untreated*
HN 056	HPV positive	BOT	7	cT2N2c	IVa	Biopsy	Untreated
HN 061	HPV positive	R. tonsil	7	cT2N2a	IVa	Biopsy	Untreated
HN 062	HPV positive	BOT	7	cT4N2c	IVa	Biopsy	Untreated
HN 064	HPV positive	R. tonsil	7	cT1N2b, pT3N2b	IVa	Biopsy	Untreated
HN 065	HPV positive	Tonsil	7	cT4bN2c	IVb	Biopsy	Untreated
HN 084	HPV positive	Tonsil	7	cT2N2b, pT2N2b	IVa	Resection	Untreated
HN 094	HPV positive	Tonsil	7	cT2N2b	IVa	Biopsy	Untreated
HN 121	HPV positive	Tonsillar pillar	7	cT1N1, pT1N0	I	Resection	Untreated
HN 161	HPV positive	Oropharynx	7	cT2N3	IVb	Biopsy	Untreated
HN 370	HPV positive	BOT	7	cT1N1, pT1NX	III	Resection	Untreated
DCA 015-1237	HPV negative	Tonsil	7	cT4aN1	IVa	Biopsy	Untreated
HN 003	HPV negative	R. tonsil	6	cT2N2b	IVa	Biopsy	Untreated
HN 013	HPV negative	Oropharynx	6	cT1N0	I	Biopsy	Untreated
HN 026	HPV negative	Oropharynx	6	cT3N2b	IVa	Resection	Untreated
HN 053	HPV negative	R. tonsil	7	cT1N2c, pT1N2a	IVa	Resection	Untreated
HN 057	HPV negative	R. tonsil	7	cT4aN2b, pT4aN2b	IVa	Biopsy	Untreated
HN 169	HPV negative	R. tonsil	7	cT1N0, pT1N0	I	Resection	Untreated
HN 185	HPV negative	Tonsil	7	cT2N0, pT1NX	II	Resection	Untreated
HN 232	HPV negative	Tonsil	7	cT1N2b, pT2N2b	IVa	Resection	Untreated
HN 235	HPV negative	BOT	7	cT1N2b, pT1N2b	IVa	Resection	Untreated
HN 253	HPV negative	BOT, FOM	7	cT2N0, pT2NX	II	Resection	Untreated
HN 310	HPV negative	Tonsillar pillar	7	cT2N2b, pT1N2b	IVa	Resection	Untreated
09-26815	Normal tonsil	Tonsil					

19x38734	Normal tonsil	Tonsil
10-11318	Normal tonsil	Tonsil
09-25936	Normal tonsil	Tonsil
18x05001	Normal tonsil	Tonsil
18x40899	Normal tonsil	Tonsil
19x40087	Normal tonsil	Tonsil
19x45743	Normal tonsil	Tonsil
19x40646	Normal tonsil	Tonsil
19x40648	Normal tonsil	Tonsil
19x46251	Normal tonsil	Tonsil
19x44612	Normal tonsil	Tonsil

AJCC, American Joint Committee on Cancer; TNM, Tumor Node Metastasis; cTNM, clinical staging; pTNM, pathologic staging; BOT, Base of tongue; R. Tonsil, Right Tonsil; FOM, Floor of Mouth; CRT, chemoradiotherapy

Table S2. CRISPR screen library preparation primers.

Name	Sequence 5'→3'
P5 0 nt stagger	AATGATACGGCGACCACCGAGATCTACACTCTTTCCCTACACGACGCTCTTCCGATCTTTGTGGAAAGGACGAAACACCG
P5 1nt stagger	AATGATACGGCGACCACCGAGATCTACACTCTTTCCCTACACGACGCTCTTCCGATCTCTTGTGGAAAGGACGAAACACCG
P5 2 nt stagger	AATGATACGGCGACCACCGAGATCTACACTCTTTCCCTACACGACGCTCTTCCGATCTGCTTGTGGAAAGGACGAAACACCG
P5 3 nt stagger	AATGATACGGCGACCACCGAGATCTACACTCTTTCCCTACACGACGCTCTTCCGATCTAGCTTGTGGAAAGGACGAAACACCG
P5 4 nt stagger	AATGATACGGCGACCACCGAGATCTACACTCTTTCCCTACACGACGCTCTTCCGATCTCAACTTGTGGAAAGGACGAAACACCG
P5 6 nt stagger	AATGATACGGCGACCACCGAGATCTACACTCTTTCCCTACACGACGCTCTTCCGATCTTGCACCTTGTGGAAAGGACGAAACACCG
P5 7nt stagger	AATGATACGGCGACCACCGAGATCTACACTCTTTCCCTACACGACGCTCTTCCGATCTACGCAACTTGTGGAAAGGACGAAACACCG
P5 8nt stagger	AATGATACGGCGACCACCGAGATCTACACTCTTTCCCTACACGACGCTCTTCCGATCTGAAGACCCTTGTGGAAAGGACGAAACACCG
P7_D701	CAAGCAGAAGACGGCATACGAGATATTACTCGGTGACTGGAGTTCAGACGTGTGCTCTTCCGATCTTCTACTATTCTTTCCCCTGCACTGT
P7_D702	CAAGCAGAAGACGGCATACGAGATTCCGGAGAGTGACTGGAGTTCAGACGTGTGCTCTTCCGATCTTCTACTATTCTTTCCCCTGCACTGT
P7_D703	CAAGCAGAAGACGGCATACGAGATCGCTCATTGTGACTGGAGTTCAGACGTGTGCTCTTCCGATCTTCTACTATTCTTTCCCCTGCACTGT

P7_D704	CAAGCAGAAGACGGCATACGAGATGAGATTCCGTGACTGGAGTTCAGACGTGTGCTCTTCCGATCTTCTACTATTCTTTCCCCTGCACTGT
P7_D705	CAAGCAGAAGACGGCATACGAGATATTCAGAAGTGACTGGAGTTCAGACGTGTGCTCTTCCGATCTTCTACTATTCTTTCCCCTGCACTGT
P7_D706	CAAGCAGAAGACGGCATACGAGATGAATTCGTGTGACTGGAGTTCAGACGTGTGCTCTTCCGATCTTCTACTATTCTTTCCCCTGCACTGT
P7_D707	CAAGCAGAAGACGGCATACGAGATCTGAAGCTGTGACTGGAGTTCAGACGTGTGCTCTTCCGATCTTCTACTATTCTTTCCCCTGCACTGT
P7_D708	CAAGCAGAAGACGGCATACGAGATTAATGCGCGTGACTGGAGTTCAGACGTGTGCTCTTCCGATCTTCTACTATTCTTTCCCCTGCACTGT
P7_D709	CAAGCAGAAGACGGCATACGAGATCGGCTATGGTGACTGGAGTTCAGACGTGTGCTCTTCCGATCTTCTACTATTCTTTCCCCTGCACTGT
P7_D710	CAAGCAGAAGACGGCATACGAGATTCCGCGAAGTGACTGGAGTTCAGACGTGTGCTCTTCCGATCTTCTACTATTCTTTCCCCTGCACTGT
P7_D711	CAAGCAGAAGACGGCATACGAGATTCTCGCGCGTGACTGGAGTTCAGACGTGTGCTCTTCCGATCTTCTACTATTCTTTCCCCTGCACTGT
P7_D712	CAAGCAGAAGACGGCATACGAGATAGCGATAGGTGACTGGAGTTCAGACGTGTGCTCTTCCGATCTTCTACTATTCTTTCCCCTGCACTGT

nt, nucleotide

Table S3. Top gene hits from CRISPR screens.

Cell Line	Pathway	Gene	p-value	Log2FC
SCC90	Autophagy	DAPK2	0.013896	-2.9161
		PRKAG3	0.0095392	-1.0037
		PPP2R1A	0.023097	-1.2411
		PPP2R2A	0.0015497	-1.2247
		STK11	0.013681	-1.19
		ATG101	5.26E-05	-1.0583
		ATG4D	0.0020869	-1.5723
		SEC62	0.028191	-1.2075
		TMEM127	2.59E-07	-2.4303
		WWP2	0.033449	-1.2832
	Autophagosome Formation	ARPC4	0.017569	-1.1626
		FFAR1	0.011026	-1.0298
		GNRHR	0.016507	-2.6035
		PIP4K2C	0.011034	-1.138
		PLA2G4C	0.0041237	-1.2444
		PTGER4	0.0097847	-1.8671
		SLC52A1	0.0068589	-1.2422
		TLR7	0.010218	-1.3321
	Phagosome Maturation	VPS16	0.04047	-1.3148
		VPS33A	9.92E-05	-1.1801
		ATP6V0C	0.0172	-1.6021
		ATP6V1B2	0.035492	-4.1933
		ATP6V1F	0.03834	-1.1905
		ATP6V1G1	0.012223	-1.6496
		GPAA1	0.011542	-1.4366
		PRDX1	0.0079084	-1.2382
		VAMP2	0.010068	-1.5164
	Ubiquitination	DNAJC1	0.0051675	-1.2736
		PSMD1	0.027994	-2.1084
		STUB1	0.02808	-1.344
		UBE2R2	0.020954	-1.0998
SCC152	Autophagy	ATG2A	0.040818	-3.0706
		EGF	0.048041	-6.9005
		GNB1L	0.012132	-7.7967
		IRS1	0.02402	-6.5304
		KAT5	0.019559	-6.8592
		PPP2R3B	0.012641	-3.5924
		RB1CC1	0.043445	-5.2359
		TNFRSF1A	0.036252	-6.5676
		ULK1	0.039791	-7.4647
	Autophagosome Formation	ADGRB1	0.039301	-4.3287
		ADGRF1	0.01049	-6.4615
		ADORA2B	0.02659	-1.042
		BDKRB1	0.044171	-1.3841
		CMKLR1	0.0055335	-4.5932
		EDNRA	0.012393	-1.5534
		ERAS	0.014424	-4.4714

	FCER2	0.015581	-5.709
	GCGR	0.048985	-4.5256
	GRM5	0.023112	-1.4277
	HRH3	0.011973	-7.4434
	HTR1B	0.017706	-6.6624
	HTR4	0.029348	-6.9309
	ITGAM	0.031611	-6.2632
	MYH8	0.031114	-5.3139
	MYO18A	0.042658	-6.4235
	NTSR2	0.021027	-6.6841
	PLA2G10	0.04484	-6.8127
	PLA2G3	0.0099765	-6.1127
	PNPLA2	0.021513	-6.2992
	TACR3	0.036679	-6.4943
	TAS1R1	0.049322	-3.8199
	TIMD4	0.0090075	-6.2743
	VIPR2	0.017774	-6.4525
Phagosome Maturation	ATP6V1G2	0.03302	-4.1875
	DYNC1I1	0.043059	-5.7985
	HLA-DRB1	0.031877	-6.3915
	MR1	0.021497	-6.2588
	NCF2	0.023211	-7.0853
	RAB5B	0.012911	-7.3885
	TUBA8	0.018503	-5.7581
	VAMP3	0.04848	-6.5655
	VPS37B	0.011603	-7.6175
Ubiquitination	HSPA4L	0.039565	-1.4248
	PAN2	0.017905	-5.9681
	PSMC5	0.042869	-7.1403
	PSMD13	0.0055735	-8.1315
	PSMD14	0.034297	-6.826
	PSMD4	0.022724	-7.0386
	UBE3A	0.00096061	-8.2309
	UCHL3	0.011234	-6.0682
	USP14	0.014213	-6.4437
	USP20	0.01219	-7.7945
	USP39	0.033652	-7.6274
	XIAP	0.026537	-6.0412

Log2FC, Log_2(Fold Change)

Table S4. sgRNA oligos

Name	Sequence 5' → 3'
RB1CC1 sg1 Fwd	CACCGTTTCTAACAGCTCTATTACG
RB1CC1 sg1 Rev	AAACCGTAATAGAGCTGTTAGAAAC
RB1CC1 sg2 Fwd	CACCGCTGGTTAGGCACTCCAACAG
RB1CC1 sg2 Rev	AAACCTGTTGGAGTGCCTAACCAGC
ATG13 sg1 Fwd	CACCGTTTACCCAATCTGAACCCGT

ATG13 sg1 Rev	AAACACGGGTTCAGATTGGGTAAAC
ATG13 sg2 Fwd	CACCGATGTGAACTCACCTACTGGA
ATG13 sg2 Rev	AAACTCCAGTAGGTGAGTTCACATC
NRBF2 sg1 Fwd	CACCGCTCAGGCAGGCATTTCTCTG
NRBF2 sg1 Rev	AAACCAGAGAAATGCCTGCCTGAGC
NRBF2 sg2 Fwd	CACCGTGAGGAGGAGCTGTTTCATA
NRBF2 sg2 Rev	AAACTATGAAACAGCTCCTCCTCAC
PIK3C3 sg1 Fwd	CACCGATACACATCCCATATGGTGA
PIK3C3 sg1 Rev	AAACTCACCATATGGGATGTGTATC
PIK3C3 sg2 Fwd	CACCGTAACTTACCATAGACATCTG
PIK3C3 sg2 Rev	AAACCAGATGTCTATGGTAAGTTAC
ATG14 sg1 Fwd	CACCGAGGAAGTAAAGACGGGTGTG
ATG14 sg1 Rev	AAACCACACCCGTCTTTACTTCCTC
ATG14 sg2 Fwd	CACCGCAGCACTGATGGTGTAGGCA
ATG14 sg2 Rev	AAACTGCCTACACCATCAGTGCTGC
NDP52 sg1 Fwd	CACCGCAGCAGGAAGTCCAATTCAA
NDP52 sg1 Rev	AAACTTGAATTGGACTTCCTGCTGC
NDP52 sg2 Fwd	CACCGTCAGGTCATCTTTAACAGTG
NDP52 sg2 Rev	AAACCACTGTTAAAGATGACCTGAC
SQSTM1 sg1 Fwd	CACCGCCTCACCTGATTCTGCCGTG
SQSTM1 sg1 Rev	AAACCACGGCAGAATCAGGTGAGGC
SQSTM1 sg2 Fwd	CACCGTGGCTCCGGAAGGTGAAACA
SQSTM1 sg2 Rev	AAACTGTTTCACCTTCCGGAGCCAC
NBR1 sg1 Fwd	CACCGTCTGTGTCACATGGAACAAG
NBR1 sg1 Rev	AAACCTTGTTCCATGTGACACAGAC
NBR1 sg2 Fwd	CACCGATGATACTGCACCAGACCCG
NBR1 sg2 Rev	AAACCGGGTCTGGTGCAGTATCATC
ATG5 sg1 Fwd	CACCGTGATATAGCGTGAAACAAGT
ATG5 sg1 Rev	AAACACTTGTTTCACGCTATATCAC
ATG5 sg2 Fwd	CACCGCCTTAGATGGACAGTGCAGA
ATG5 sg2 Rev	AAACTCTGCACTGTCCATCTAAGGC
ATG7 sg1 Fwd	CACCGTCCTACTTTAGACTTGGACA
ATG7 sg1 Rev	AAACTGTCCAAGTCTAAAGTAGGAC
ATG7 sg2 Fwd	CACCGCTCTTGTAAATACCATCTGT
ATG7 sg2 Rev	AAACACAGATGGTATTTACAAGAGC
TAP1 sg1 Fwd	CACCGCATCATGTCTCGGGTAACAG
TAP1 sg1 Rev	AAACCTGTTACCCGAGACATGATGC
TAP1 sg2 Fwd	CACCGGGCTCCAAGAGCGAAAACGC
TAP1 sg2 Rev	AAACGCGTTTTCGCTCTTGGAGCCC
TAP2 sg1 Fwd	CACCGGTTGATTCGAGACATGGTGT
TAP2 sg1 Rev	AAACACACCATGTCTCGAATCAAC
TAP2 sg2 Fwd	CACCGATCCCCATATATGTATACCA
TAP2 sg2 Rev	AAACTGGTATACATATATGGGGATC
TAPBP sg1 Fwd	CACCGGATCGAGTGTTGGTTCGTGG
TAPBP sg1 Rev	AAACCCACGAACCAACACTCGATCC
TAPBP sg2 Fwd	CACCGAAGCGGCTCATCTCGCAGTG
TAPBP sg2 Rev	AAACCACTGCGAGATGAGCCGCTTC
Mouse Ulk1 sg2 Fwd	CACCGCGGCCCGCTGAAGACACCCG
Mouse Ulk1 sg2 Rev	AAACCGGGTGTCTTCAGCGGGCCGC
Mouse *Ulk1* sg3 Fwd	CACCGTAGTCTGCGTACCACTAGGG
Mouse *Ulk1* sg3 Rev	AAACCCCTAGTGGTACGCAGACTAC

Fwd, forward primer; Rev, reverse primer

Table S5. TIDE analysis primers.

Name	Sequence 5'→3'
RB1CC1 sg1 Fwd	TGGTGTTGCTTTGTAATGCTTC
RB1CC1 sg1 Rev	GAATTCAACTTGCATACCTCCC
RB1CC1 sg2 Fwd	ATGGAGAGGTGGTGAGATTTGT
RB1CC1 sg2 Rev	AGAGAGCACCAGTTCAGTGGAT
ATG13 sg1 Fwd	AGACTGTCCAAGTGATTGTCCA
ATG13 sg1 Rev	CACAGCAGAAAGTTAAGAACCAAA
ATG13 sg2 Fwd	GCTAGAATGTGAAGTTCCCCTC
ATG13 sg2 Rev	TAAATAAGCACGTGTGTCAGGG
NRBF2 sg1 Fwd	AGCGTGAAGAAAGATTGAAAGC
NRBF2 sg1 Rev	TGTTTTATCATCTTTTGGGGCT
NRBF2 sg2 Fwd	GTGTTGGGTTATTCACAAAGGA
NRBF2 sg2 Rev	GCTTTCAATCTTTCTTCACGCT
PIK3C3 sg1 Fwd	GGAATGAATGGCTGAAACTACC
PIK3C3 sg1 Rev	GAAAAGGGTCAGAAAGCTGCTA
PIK3C3 sg2 Fwd	TGCTCTGTAATCTAGGACGGTG
PIK3C3 sg2 Rev	AAATTCCATCAGAAACAGTGCC
ATG14 sg1 Fwd	ATAATCGCAAACTTGGTGACCT
ATG14 sg1 Rev	CAGGAAAACCAATGACCCTAGA
ATG14 sg2 Fwd	TTATGTCTGGTCATGGAGCATC
ATG14 sg2 Rev	TTCAATTACTTGCCTGTTGCAG
NDP52 sg1 Fwd	ACCTTCATGTGGGTTACTTTGC
NDP52 sg1 Rev	CATCCTCATCCACATAGCAGAA
NDP52 sg2 Fwd	GGATTTTGCTTTTCCTGACTTG
NDP52 sg2 Rev	ATGAAATGCTGGGTGAAGGTAT
SQSTM1 sg1 Fwd	GAAGGTGAAACACGGACACTTC
SQSTM1 sg1 Rev	GGTATCCTGAATTCTTGCCTTG
SQSTM1 sg2 Fwd	CTACGACTTGTGTAGCGTCTGC
SQSTM1 sg2 Rev	AGTTTCCTGGTGGACCCATT
NBR1 sg1 Fwd	GGAGCAGGCTAGAGACTTTGTT
NBR1 sg1 Rev	AGTTAAAACCCAAGCGAGACAG
NBR1 sg2 Fwd	TGTATCTGTGGAGTTCATTGCC
NBR1 sg2 Rev	TGTCAGTCAATGCTCACCTCTT
TAP1 sg1 Fwd	CTCATCACTTGGAACCTGTCTG
TAP1 sg1 Rev	GGTACCATTTTCCCACCTTCTT
TAP1 sg2 Fwd	AGTACTGCTACTTCTCGCCGAC
TAP1 sg2 Rev	ATGAGATCAGCTCTCGGAACA
TAP2 sg1 Fwd	CATCTCCCTCCCCTCTTATTCT
TAP2 sg1 Rev	TTAGTCTCCTGGAAGAAACCGA
TAP2 sg2 Fwd	CAAATTGGAACACTGGGGTATT
TAP2 sg2 Rev	GTCGGTCCATGTAGGAGAAAAC
TAPBP sg1 Fwd	GCAGGTCACCAGACATACAAAC
TAPBP sg1 Rev	ACTGAGATAGAGCTCAGGGTCG
TAPBP sg2 Fwd	TCCTTCTCTACACTCAGACCCC
TAPBP sg2 Rev	ATATGCTGACCATCAGCCAAG
Mouse *Ulk1* sg2 Fwd	GGGGTAGTAATGACACCACCTC
Mouse *Ulk1* sg2 Rev	ACTTCTCGAATCTCCCAAACAA

Fwd, forward primer; Rev, reverse primer

Table S6. TIDE analysis results

Cell Line	sgRNA	TIDE efficiency
SCC90	ATG13 sg1	76.8
	ATG13 sg2	81.7
	ATG14 sg1	90.6
	ATG14 sg2	98.0
	TAPBP sg1	86.9
	TAPBP sg2	96.0
SCC152	ATG13 sg1	75.1
	ATG13 sg2	86.8
	ATG14 sg1	92.3
	ATG14 sg2	56.8
	RB1CC1 sg1	44.1
	RB1CC1 sg2	82.1
	PIK3C3 sg1	90.4
	PIK3C3 sg2	85.5
	NRBF2 sg2	75.1
	NDP52 sg1	83.6
	NDP52 sg2	77.4
	SQSTM1 sg1	87.3
	SQSTM1 sg2	98.3
	NBR1 sg1	76.2
	NBR1 sg2	78.5
	TAPBP sg1	80.1
	TAPBP sg2	93.5

TIDE, Tracking of Indels by DEcomposition

Table S7. Antibodies

Antibody	Reactivity	Source	Catalog	Clone No.	RRID
HLA Class I ABC	Human	ProteinTech	15240-1-AP		AB_1557426
HLA Class I ABC	Human	ProteinTech	66013-1-1g	5C5B7	AB_11042593
Cytokeratin (pan-reactive)	Human	BioLegend	628604	C-11	AB_2563652
LC3B I/II	Human, Mouse	Cell Signaling	2775S		AB_915950
ATG14/Barkor	Human, Mouse	ProteinTech	19491-1-AP		AB_10642701
Phospho-ATG14 (Ser29)	Human, Mouse	Cell Signaling	92340	D4B8M	AB_2800182
β-actin	Human, Mouse	BioLegend	664804	W16197A	AB_ 2728496
ULK1	Human, Mouse	Cell Signaling	8054	D8H5	AB_11178668
NBR1	Human, Mouse	ProteinTech	16004-1-AP		AB_2251178
P62	Human	ProteinTech	18420-1-AP		AB_10694431
NDP52	Human	ProteinTech	12229-1-AP		AB_11182600
Calreticulin	Human, Mouse	ProteinTech	27298-1-AP		AB_2880835
HLA-A, B, C FITC	Human	BioLegend	311404	W6/32	AB_314873
CD11b eFLuor 450	Mouse	eBioScience	48-0112-80	M1/70	AB_1582236
Ly6C BV510	Mouse	BioLegend	128033	HK1.4	AB_2562351
F4/80 BV605	Mouse	BioLegend	123133	BM8	AB_2562305
CD3 BV711	Mouse	BioLegend	100241	17A2	AB_2563945
CD45 PerCP	Mouse	BioLegend	103129	30-F11	AB_893343
I-A/I-E PE	Mouse	BioLegend	107608	M5/114.15.2	AB_313323
NKp46 PE/Cy7	Mouse	BioLegend	137617	29A1.4	AB_11219186
CD19 APC	Mouse	BioLegend	115512	6D5	AB_313647
Ly6G AF700	Mouse	BioLegend	127621	1A8	AB_10643269

CD4 FITC	Mouse	BioLegend	100510	RM4-5	AB_312713
CD8a PE/Cy7	Mouse	BioLegend	100722	53-6.7	AB_312761
CD62L AF700	Mouse	BioLegend	104426	MEL-14	AB_493719
CD44 PE	Human, Mouse	BioLegend	103007	IM7	AB_312958
CD28	Mouse	BioLegend	102102	37.51	AB_312867

RRID, Research Resource Identifier
